# The SNARC Effect in Number Memorization and Retrieval. What is the Impact of Congruency, Magnitude and the Exact Position of Numbers in Short-Term Memory Processing?

**DOI:** 10.5709/acp-0198-0

**Published:** 2016-12-31

**Authors:** Małgorzata Gut, Rafał Staniszewski

**Affiliations:** 1 Faculty of Humanities, Nicolaus Copernicus University, Toruń, Poland; 2 Department of Cognitive Psychology, University of Finance and Management, Warsaw, Poland; 3 Progreso, Center for Support of Development, Warsaw, Poland

**Keywords:** SNARC effect, Mental Number Line, short-term memory, retrieval

## Abstract

Mental representations of numbers are spatially organized along a Mental Number Line (MNL). One widely proven manifestation of this relationship is the Spatial Numerical Association of Response Codes (SNARC) effect. It refers to the phenomenon of faster responses to numbers when there is congruency between the reaction side and the number position on the MNL . Although long-term memory is considered to house the MNL, short-term memory (STM) load may also modulate responses to numbers and the SN ARCRC effect. Our question, however, was not how STM content modulates the SNARC effect observed in responses to digits, but rather how the MNLNL representation affects the number retrieval from ST M. Each trial began with four digits presented horizontally in a spatial sequence (prime stimuli), which were then replaced by one of the priming digits as a single target. The task required participants to recall the exact location of the target. The SN ARCRC effect occurred only in the retrieval of left-sided digits, most likely because of the generally better processing of right-sided ones, as well as in reaction to digits presented more laterally. Moreover, memory processing was more efficient with low-magnitude numbers, which may suggest that they trigger attention shifting. We conclude that the MNL affects not only the responses to numbers obtained in typical SNARC-induction tasks, such as number detection, parity judgment or magnitude comparison, but also memorization and retrieval of them. Importantly, this effect seems to be dependent on the exact position of a digit in STM.

## Introduction

 The connection between numerical cognition and spatial cognition has been demonstrated in a number of behavioural as well as clinical studies (for a review see Fias & Fischer, 2005; Hubbard, Piazza, Pinel, & Dehaene, 2005). A clear example of this relationship is the Spatial Numerical Association of Response Codes (SNARC) effect (Dehaene, Bossini, & Giraux, 1993). This effect refers to an association between numerical magnitude and the side of response. Participants’ responses to larger magnitude numbers are faster with their right than with their left hand, and their responses to smaller numbers are faster with their left hand than the right. This effect has been replicated many times with the use of different paradigms and types of stimuli or tasks (for a review see Fias & Fischer, 2005; Fias, van Dijck, & Gevers, 2011; Fischer & Shaki, 2014; Wood, Nuerk, Willmes, & Fischer, 2008), and it occurs even when the processed number magnitudes are completely irrelevant to the task (e.g., Fias, Brysbaert, Geypens, & d’Ydewalle, 1996; Fias, Lauwereyns, & Lammertyn, 2001; Lammertyn, Fias, & Lauwereyns, 2002). 

 The SNARC effect is also independent of the stimuli’s modality (Fischer, Shaki, & Cruise, 2009; Nuerk, Wood, & Willmes, 2005) and of the number rotation (Ganor-Stern & Tzelgov, 2008; Nuerk et al., 2005; Reynvoet & Brysbaert, 2004), as it has been observed for visual and auditory stimuli as well as for Arabic digits, word numerals, or dots in arrays. This effect has been interpreted in terms of a theory of the analogue representations of magnitudes, which is associated with the concept of the Mental Number Line (MNL), on which numbers are spatially organized from left to right according to their numerical magnitudes (Dehaene, 1992). These representations are stored in long-term memory (LTM), which may suggest that this type of LTM-dependent information is responsible for eliciting the SNARC effect. However, a substantial amount of research has shown that short-term memory (STM) processing plays an important role in the generation and/or modulation of this spatial-numerical relationship (for a review see Fias et al., 2011). The SNARC effect has been studied with the use of a variety of tasks and types of stimuli. However, the most common methods, which induce this spatial-numerical association in reaction times (RTs) and correctness of responses, are parity judgment and magnitude comparison tasks. Other methods reported in the literature are based on stimulus detection, line bisection, pointing (related to, e.g., assessment of the number location on the line flanked by reference numbers), random number generation, or counting (for a review see Fischer & Shaki, 2014). Surprisingly, there are no reports concerning the use of number recall rates as an indicator of the SNARC effect, so the question (and one of our goals) is whether these behavioral indices can be applied for this purpose. More precisely, we hypothesized that congruency between the spatial positions of all numbers in the memorized stimulus and their spatial positions on the MNL would facilitate the time and accuracy of their retrieval from STM. Thus, we assumed that it would be easier to retrieve, for example, digit 2 from STM when it had been displayed on the left side of the stimuli than when it was presented on the right side, which should be reflected in faster and more correct recalls. 

### The Impact of STM Load on the Spatial Numerical Association of Response Codes Effect

 A great range of experimental data confirms the role of working memory in formation of the spatial-numerical association. For example, the profile of this spatial-numerical link may be the result of individual strategies used in particular task requirements (Fischer, 2006). Lindemann, Abolafia, Pratt, and Bekkering (2008) investigated whether the SNARC effect was caused by the current cognitive coding strategies of each participant. The researchers modulated the SNARC effect by using a particular sequential order of digits and the activation of their representation in working memory. The participants were asked to memorize the spatial locations of three different digits presented in a horizontal arrangement. This sequence could have had an ascending order (e.g., 456), a descending order (e.g., 432), or a random order (e.g., 687). The sequence was then replaced by a single one-digit number to perform the parity judgment task (the aim of which was to reveal the SNARC effect), while simultaneously committing the spatial locations of the previously presented numbers to memory. After the parity judgment test, participants were required to recall the spatial location of one digit from the sequence. The authors assumed that the memory task would interfere with the parity judgment and that the storage of the spatial organization of the sequence would modulate the SNARC effect. Indeed, they observed an impact of STM representations on the SNARC effect assessed by the parity judgment task. More precisely, the SNARC effect was obtained only in the trials with ascending or randomly ordered sequences that were stored in memory. These findings confirm that spatial-numerical relationships are not characterized by automatic cognitive processing. Instead, memory load and the strategies responsible for coding digits in STM drive the SNARC effect. 

 Moreover, it is possible that numbers and space have no intrinsic and obligatory relationship, and that rather this association is constructed during an experiment on the basis of the task’s instructions or the context of stimuli during the performance of the task. This would mean that the spatial-numerical association is more modifiable than expected if the LTM-related representations were the sole driver of the relationship. For example, the relationship between a particular number and the side of response is dependent on the range of numbers used in the experiment (Dehaene et al., 1993) and the magnitude of a reference number (Ben Nathan, Shaki, Salti, & Algom, 2009). Others have demonstrated that the SNARC effect can be easily modulated by task instruction, for example, imagining numbers on a clock face versus a ruler (Bächtold, Baumüller, & Brugger, 1998), and by the activity preceding the SNARC task, for example, scanning text written in a language that requires a particular direction of reading (Shaki & Fischer, 2008). All of these facts seem to indicate that the relationship between numbers and space is created during a task, which means that STM and its contents have an important contribution to the SNARC effect profile (i.e., whether the SNARC effect is clearly pronounced, rather indistinct, or even reversed). The impact of STM has also been tested through the use of a dual-task procedure (Herrera, Macizo, & Semenza, 2008). In that study, participants completed the magnitude comparison task in a single-comparison condition (without any additional task) or in a dual-task condition (with an additional task that required storing a specific type of information in memory for later recall). The authors concluded that the SNARC effect could be modulated or suppressed under the conditions of working memory load. The spatial-numerical association thus depends on the availability of STM resources and the type of information that has to be memorized. 

### The Effect of Item Ordinal Position in Short-Term Memory on the Spatial-Numerical Association

 The order of the objects that are displayed to be memorized could also be essential to the formation of a spatial character of number representations. A study by van Dijck and Fias (2011) showed that mental associations with spatial locations (left/right) were related to the serial position of an item in working memory, which has been called the Ordinal Position Effect (OPE). This effect suggests that stimuli that are positioned at the beginning of a sequence stored in STM are associated with the left side and subsequently receive faster response with the left hand, similarly for the right side and items placed in memory at the end of the stimuli sequence. Consequently, in their study, the magnitude of the numbers (and their position on the MNL) was not associated with the side of the response. Furthermore, they found that the OPE could be obtained in the subsequent choice response task for not only numbers but for any type of stimuli stored in STM; the stimuli could even be fruit and vegetable names. That is to say, the vegetable and fruit names presented at the beginning of the sequence (and stored in STM first) were mentally associated with the left side, which resulted in faster left-side responses to them. On the contrary, any words presented at the end of the temporal sequence were mentally linked to the right side and resulted in faster right-side reactions. 

 The role of ordinal information (not the effect of stimuli appearance order but rather the mental representation of ordering) in evoking the spatial-numerical relationship has also been discussed in the context of the studies focused on SNARC-like effects, observed for letters, names of the days of the week, and names of the months (Gevers, Reynvoet, & Fias, 2004). Although such a type of information is not quantitative per se, it has been argued that names of months or letters of the alphabet can activate the spatial-numerical representations due to learned relationships between, for example, months and the numbers that represent them in the sequence (e.g., the Roman numeral III for March). However, this spatial representation of ordinal information has been recently shown for a type of material that has no reference to mental numerical representation, namely for Chinese words for color names (Zhang et al., 2016). They occur in a specific order in the color spectrum but have no spatial or numerical connotation for Chinese people. 

 The literature reviewed above clearly demonstrates that the SNARC effect is not necessarily driven by number magnitude and the MNL in LTM but rather by the relationship between space and order position of an item in working memory. This again suggests that the way in which numbers are stored in working memory is crucial for the SNARC effect. However, this finding can be questioned by the observation that the SNARC effect is also present in cases when a memory task is not performed and participants still remember the numbers in the presented order (such as during the single task in the study by Herrera et al., 2008). Fias, van Dijck, and Gevers (2011) proposed that individuals store numbers in STM as a set of stimuli, and that during the performance of the task, they order them according to their numerical magnitude to assist with memory processing, which could be a type of strategy. Also, it cannot be excluded that spatial-numerical association is related to the particular order of numbers represented in LTM, which is an effect of the way of counting and experiences with ordinal numerals. Consequently, in case of there being no additional memory task, a manner of ordering the numbers may affect the way someone memorizes number material. 

### The Distinction Between the Effects of Long-Term Memory and Short-Term Memory Representations on the Relationship Between Numbers and Space

 Van Dijck and Fias (2011) have suggested that the OPE and the SNARC effect cannot be obtained during the same task because they are mutually exclusive. However, in a recent study, Ginsburg and Gevers (2015) questioned this suggestion by demonstrating that both effects engage two different representations and that spatial-numerical associations are the result of number representations in LTM as well as the number order of the displayed material stored in STM. In their experiment, participants in each trial were required to memorize a sequence of numbers, respond to a single one-digit number in a magnitude judgment task, and finally to recall the number sequence from the beginning of the trial. Moreover, during one part of the paradigm (the “inducer” part), the participants were asked to complete a magnitude judgment task for all presented numbers, whereas during another part (the “diagnostic” part), they performed this task only for the numbers previously displayed in the sequence using a “GO/NO-GO” procedure. The aim of this procedure was to ensure that the participants would process the numbers displayed in the sequence in their working memory for the diagnostic part. The authors demonstrated that in the experimental condition, when participants reacted to all numbers in a one to nine interval (inducer task), the typical SNARC effect was visible, whereas no significant interaction between the serial position of the memorized numbers and response side (no OPE) was present. On the contrary, during the GO/NO-GO condition, the position of memorized numbers resulted in a clear OPE but no SNARC effect. Most recently Huber, Klein, Moeller, and Willmes (2016) investigated the relationship between the SNARC effect and the OPE by manipulation of the number of digits in the stored sequence as well as the number range used in the task. They confirmed the co-existence of both effects. In addition, they revealed that using the number interval from one to 10 (instead of one to nine) can reduce the SNARC effect strength. In their opinion, this is why the results obtained by Lindemann et al. (2008) and, for example, van Dijck and Fias (2011) differ: because of the specificity of two-digit numbers processing and some problems with the parity judgment of 0 (see Nuerk, Moeller, Klein, Willmes, & Fischer, 2011). 

### The Goals and Research Questions of the Present Study

 Our question was whether it would be reasonable to examine the STM impact on the SNARC effect by using a simpler paradigm and a more direct method than the ones used in the studies reviewed above. First, to study the effect of the interaction between STM and LTM representations on the SNARC effect manifested during retrieval, we proposed a task that did not evoke any interference between the SNARC and OPE, as in the study by Ginsburg and Gevers (2015). The task designed for our experiment required the retrieval of spatial positions of the digits stored in STM, all being displayed at the same time on a screen, similar to the study of Lindemann et al. (2008). We assumed that despite presenting the spatial sequence of digits (instead of temporal sequences of single digits) the cognitive effect in memory processing would be the same: generating the sequence of items to remember and to retrieve. However, the difference between other experiments and the one we developed is that we did not include an additional task to elicit the SNARC effect or modulation of the effect (e.g., the parity judgment task). In this manner, we measured the effect more directly with the use of a memory task that required only the recall of the spatial position of one digit displayed in the row of four. Moreover, our question was not how the SNARC effect (measured by parity judgment or number comparison tasks) was modulated by concurrent STM load, as that has been widely reported in the literature. We were more interested in how the stable MNL representation can modulate the way we recall and process numerical information in STM. This means that we were interested in the reverse direction of the effect by asking how the LTM representation of numbers influences the STM representations in immediate recall. The expected modulation may prove that MNL affects not only one’s response to detected numbers but also the retrieval of numbers as well. 

 To put it simply, there are three essential differences between the experimental designs used in the previous studies and our concept of studying the role of STM processing in the spatial-numerical association. We did not use the most typical task to evoke the SNARC effect, we did not use any additional memory task in the procedure (we loaded STM and evoked the SNARC effect by means of one task), and consequently, there was no STM load with the material different from numbers (as proposed by Herrera et al., 2008). These three issues become important when we take into account that the type of material stored in STM (verbal/spatial) and the type of task for evoking the SNARC effect (parity judgment/magnitude comparison) differently impact the SNARC effect’s strength. This has been demonstrated in the dual-task procedure with two types of material upload and two types of the SNARC-induction tasks by van Dijck, Gevers, and Fias (2009). 

Additionally, we were interested in whether this effect would be more pronounced in trials with digits displayed in the most lateral positions in the stimuli, which would confirm the linear nature of the SNARC effect, as well as the horizontal organization of number representations order in MNL and its influence on number retrieval proficiency. Thus, the trials enabled the involvement of both STM and LTM representations of numbers in one task and helped determine whether the SNARC effect occurs in STM when the spatial location of the digits (i.e., the representation of the particular number magnitude together with its spatial position in the stimulus) is retrieved. In other words, we were interested in whether only the exact location of each digit in the spatial sequence (independent of their congruency) determines the memory trace of all numbers composing the stimulus during retrieval or whether the number representations in the MNL (which are LTM-dependent) have an additional (or even pivotal) impact on the retrieval of the spatial localization of all elements from STM.

## Method and Participants

After providing informed consent, 28 volunteers (*M*_age_ = 26 years; age range: 19-53 years; 24 women and four men) participated in the experiment. All of the participants were healthy, had no history of neurological problems, were right-handed (their handedness was assessed by self-declaration), and had normal or corrected-to-normal vision. In addition, the participants were unaware of the purpose of the study and they took part in the experiment for course credit.

### Apparatus, Stimuli, and Presentation

The participants were comfortably seated at 60 cm in front of a computer monitor and were instructed to fixate on the centre of the screen at all times. The stimuli were presented on a 17 in. CRT screen with a resolution of 1,680 × 1,050 pixels and a 60 Hz refresh rate. The participants responded by using four keys on a standard QWERTY keyboard. The left hand was used to press the left Shift and z keys, and the right hand was used to press the / and right Shift keys. The stimulus presentation and the recording of the participants’ responses were controlled by Presentation software (v. 12.1; www.neurobs.com).

### Stimuli and Experimental Design

Per each trial, the stimuli consisted of a single centrally displayed digit (the target stimulus) or a row of 4 digits presented laterally to the centrally displayed fixation point (the prime stimuli). The single digits were presented in a black font, 16 × 10 mm in size, and extended 1.53° vertically and 0.95° horizontally. Each row of four digits was 16 × 80 mm in size, and extended 1.53° vertically and 7.64° horizontally. All of the stimuli were presented on a light grey background (RGB 150, 150, 150). The stimuli comprised the following digits: 1, 2, 4, 5, 6, 8, or 9.

**Figure 1.**
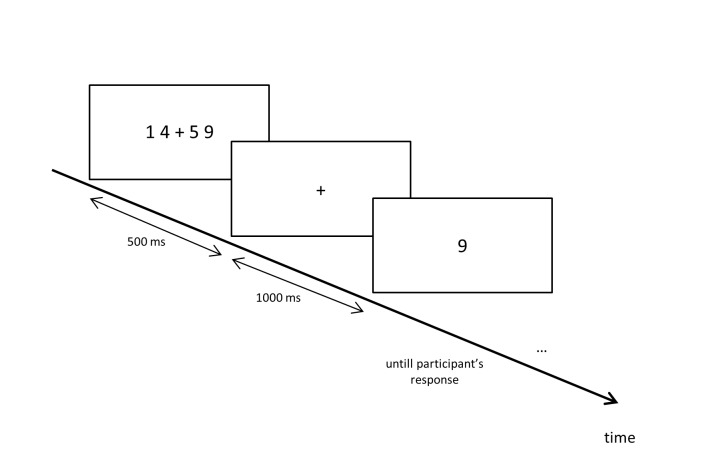
Experimental paradigm. A prime-stimulus consisting of a row of four numbers presented in four horizontal positions precedes a target stimulus, which is one of the numbers displayed in the prime. Participants are required to memorize the prime stimulus and then to determine what was the position of the single number displayed later as the target.

 Each trial started with a prime stimulus of four digits (two on the right and two on the left) that flanked the central fixation point (a black cross sign, 3 × 3 mm, extending 0.34° vertically and horizontally) and was presented for 500 ms (see Figure 1). Each digit was displayed in one of four horizontal locations among the other stimuli: laterally left, medially left (on the left side, but closer to the fixation point), medially right, and laterally right. The participants were asked to remember all of the digits and their spatial positions, and the prime stimulus was subsequently replaced by a centrally displayed fixation point for 1 s. After that, the target digit, which consisted of a single digit that had been presented previously in the prime stimulus, was displayed centrally on the screen. The participant was asked to recall the spatial location of this digit in the previously presented stimulus (laterally left, medially left, medially right, or laterally right). The digit was presented until the motor response occurred. The participants were instructed to respond as quickly and as accurately as possible by pressing the proper key (in relation to the spatial position of the target digit in the stimulus). The left Shift key corresponded to the laterally left position, the z key to the medially left position, the / key to the medially right, and the right Shift key to the laterally right. According to the MNL organization, the digits in the stimuli were defined as left/low-magnitude (1 and 2), middle (4, 5, and 6), and right/high magnitude digits (8 and 9). The use of a numerical interval from one to nine (which is often used in studies on the SNARC effect) leads to the number five being mentally located exactly in the middle. Consequently, this number is mentally processed neither as a low- nor as a high magnitude. Although the task was not to compare the numerical magnitudes (which means that the magnitude was irrelevant to the task instruction), the effect size was expected to be weaker for the number five as well as the two adjacent numbers (because of the established relationship between the number magnitude and the size of the bias of the SNARC effect). We were most interested in the effect observed for the numbers positioned in the numerical distances that were on the far left and right (e.g., similar to the study by Fischer, Castel, Dodd, & Pratt, 2003, or Gut, Szumska, Wasilewska, & Jaśkowski, 2012, which used only the numbers 1, 2, 8 and 9). 

 However, in addition to the low and high numbers, we decided to use a third group of numbers (consisting of 4, 5, and 6) called the *middle* numbers to investigate the MNL representation and the spatial pattern of the number sequence in STM when being retrieved. Based on the dependence of the SNARC effect on the relative magnitudes of the interval used in a particular task (Dehaene et al., 1993, Experiment 3), we believed that during task completion, participants would process the numbers 1 and 2 as the low numbers, 8 and 9 as the high numbers, and the numbers 4, 5, and 6 as the numbers being in the middle of this interval. The lack of numbers 3 and 7 additionally enhanced (and sharpened) this division of the stimuli into three clearly separated sets. 

A trial was defined as congruent when the side of the target digit presentation (left/right) in the row of numbers was consistent with its location on the MNL (left-right), and as incongruent in the opposite case (e.g., if the digit 2 had been presented on the right side of the prime stimulus). Thus, there were four types of trials (experimental conditions): congruent with a low-magnitude number (1 or 2 on the left side of the row of digits in the lateral or medial left position), congruent with a high magnitude number (8 or 9 in the lateral or medial right location), incongruent with a low-magnitude number (with 1 or 2 on the right side of the row of digits in the lateral or medial right position), and incongruent with a high magnitude number (8 or 9 in the lateral or medial left location). Trials with the middle magnitude numbers (4, 5 and 6) were considered *neutral* trials, and these digits were also displayed in all four locations in the prime stimuli. The experimental procedure is illustrated in Figure 1.

 The experimental session consisted of 96 trials and lasted approximately 10 min. Thirty-two of these trials were congruent, 32 were incongruent, and the others were neutral. The same number of trials had low- and high magnitude numbers used as the targets. The order of the digits in the stimuli was presented in a pseudo-random manner to exclude the possibility that two numbers of the same magnitude category were displayed on the same side of the stimulus (e.g., 2 in the medially right location and 1 in the laterally right location). The aim of this pseudo-randomization was to prevent the conceivable influence of the adjacent number magnitude on the processing and retrieval of the second digit of the pair. In other words, we assumed that the second number on the same side of the prime stimulus might contribute to a better recalling of the target if their magnitudes were compatible because the numbers were in the same category and were associated with the same side of the MNL as well as the response side, thus leading to the congruity effect (see Nuerk, Bauer, Krummenacher, Heller, & Willmes, 2005).The order of the trials was randomized between subjects. Before the experiment the participants familiarized themselves with the task and stimuli by performing a short training block of 10 trials. 

Afterwards, the data including the timing of the stimulus presentations and the recorded responses from all trials were used in the analysis of the mean reaction time (RT) and the mean percentage of correct responses (PC). Matlab software (v. 7.0.4; The MathWorks Inc., Natick, MA, 2000) was used to determine the mean RTs and PCs based on the raw data for each experimental condition on its own. The mean RTs and PCs for each participant and experimental condition were analysed using SPSS software (v.22.0.0.1).

## Results

The incorrect responses, anticipatory responses (faster than 150 ms) and delayed responses (slower than 5 s), were all treated as errors (0.01% and 0.09% of all motor reactions, respectively) and were not included in the RT analyses.

To examine the effect of number magnitude and of the side and exact location (position) of the digit presentation on the mean RTs and PCs, the data were submitted to a three-factor repeated-measures analysis of variance (ANOVA). We used the Magnitude (3), Position (2), and Side of Stimuli Presentation (2) as the within-subject factors and the RTs and PCs as the dependent variables. The mean response error rate was 8.6% and the mean RT was 901 ms.

### Reaction Times

In terms of RT, the three-factor ANOVA, with Magnitude (3), Position (2), and Side of Stimuli Presentation (2) as the factors, revealed that there was a significant effect of magnitude, *F*(2, 27) = 8.759, *p* < .01, η_p_^2^ = .25, with significant differences (Bonferroni correction, used for all post hoc analysis) between the RTs for low-magnitude digits (888 ms) and high magnitude ones (926 ms), *t*(27) = 2.84, *p* < .05, as well as between the RTs for low and middle digits (925 ms), *t*(27) = 3.88, *p* < .01. There was also a significant main effect of side of stimuli presentation, *F*(1, 27) = 7.68, *p* < .05, η_p_^2^ = .22, with a slower mean RT for the digits displayed on the left side (936 ms) than for those displayed on the right (866 ms). Additionally, position had a significant main effect, *F*(1, 27) = 20.37, *p* < .01, η_p_^2^ = .43, with faster responses to the digits presented more laterally in the stimuli (873 ms) than for those displayed medially (929 ms). Moreover, a significant interaction was found between magnitude and side of stimuli presentation, *F*(1, 27) = 15.11, *p* < .01, η_p_^2^ = .36, between magnitude and position, *F*(2, 27) = 3.28, *p* < .05, η_p_^2^ = .11, and between position and side of stimuli presentation, *F*(1, 27) = 5.49, *p* < .05, η_p_^2^ = .17, and a three-factor interaction of Magnitude × Position × Side of Stimuli Presentation, *F*(1, 27) = 3.45, *p* < .05, η_p_^2^ = .11, was also significant.

### Percentage of Correct Responses

Similarly, the ANOVA for the PCs demonstrated a significant effect of magnitude, *F*(2, 27) = 6.68, *p* < .01, η_p_^2^ = .20, and side of stimuli presentation, *F*(1, 27) = 7.80, *p* < .01, η_p_^2^ = .22. The PC was significantly greater for the middle (91.25%) than for the high (90.17%) magnitude numbers, *t*(27) = 2.32, *p* < .05, and the PCs were greater for the digits displayed on the right (93.04%) than on the left (89.7%). None of the remaining differences reached significance. We also observed significant interactions between magnitude and side of stimuli presentation, *F*(1, 27) = 3.76, *p* < .05, η_p_^2^ = .12, between magnitude and position, *F*(2, 27) = 3.38, *p* < .05, η_p_^2^ = .11, and a three-factor interaction between all of these factors, *F*(2, 27) = 8.23, *p* < .01, η_p_^2^ = .23.

### Interaction Between Magnitude and Position Factors

**Figure 2.**
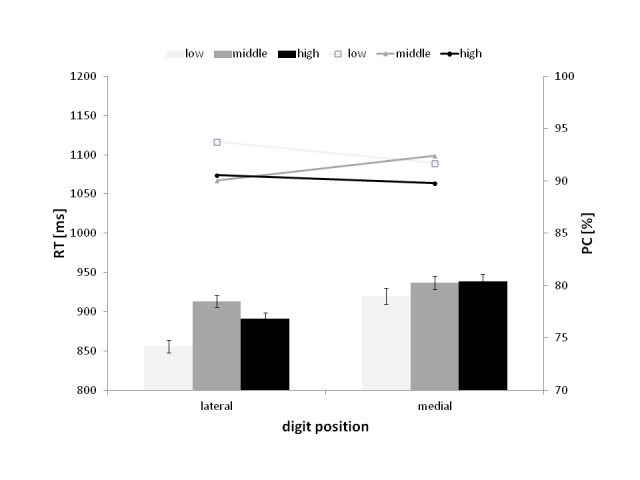
The interaction between the magnitude and the position of the digit in the mean reaction time (RT; represented by bars) and the percentage of correct responses (PC; represented by lines). The error bars represent the CI from normalized data, with Morey correction (see Morey, 2008).

As illustrated in Figure 2, which presents the Magnitude × Position interaction, the mean RT for the low-magnitude numbers (856 ms) was faster than for the high magnitude ones (891 ms), *t*(27) = 3.14, *p* < .01, and middle-magnitude numbers (913 ms), *t*(27) = 5.19, *p* < .01, for laterally presented digits, whereas digits that were displayed medially in the stimuli did not show a significant difference in mean RTs.

The pattern of the differences between the mean PCs was similar for numbers presented laterally but not for those presented medially. Specifically, for digits that were presented laterally, there was a significant difference between the PCs for low-magnitude numbers (93.73%) and high ones (90.57%), *t*(27) = 2.48, *p* <.05, and between low- and middle- (90.07%) magnitude numbers, *t*(27) = 2.59, *p* < .05. In contrast, in the trials with medially presented digits, there was only a significant difference between the PCs for high (89.77%) and middle-(92.43%) magnitude numbers, *t*(27) = 2.96, *p* < .01.

### Interaction Between Magnitude and Side of Presentation Factors

**Figure 3.**
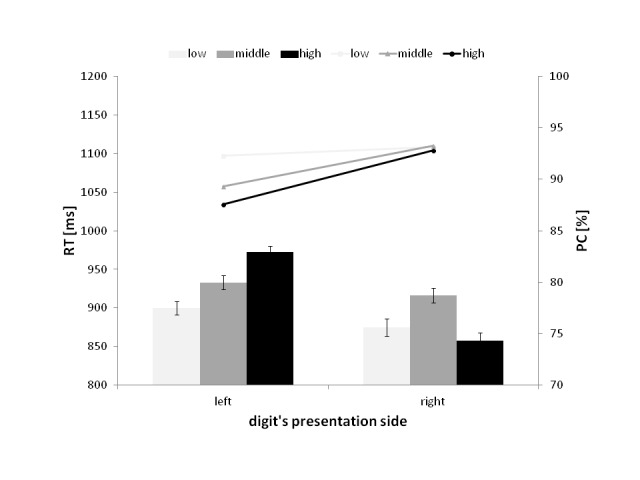
The interaction between the magnitude and the presentation side of the digit in the mean reaction time (RT; represented by bars) and the percentage of correct responses (PC; represented by lines). The error bars represent the CI from normalized data.

As shown in Figure 3, which illustrates the Magnitude × Side of Presentation interaction, when the numbers were displayed on the left, the RTs were faster for low-magnitude digits (900 ms) than for middle- (933 ms), *t*(27) = 2.58, *p* < .01, and high- (972 ms) magnitude digits, *t*(27) = 5.95, *p* < .01. Additionally, the difference between the RTs for the middle- and high magnitude numbers was significant, *t*(27) = 3.23, *p* < .01. For numbers with a right-side presentation, the difference between the RTs for low- (875 ms) and high magnitude numbers (856 ms) was not significant. However, the participants responded faster to low- than to middle-magnitude numbers (916 ms), *t*(27) = 2.76, *p* < .05. Moreover, the difference between the RTs for high magnitude and middle-magnitude ones was significant, *t*(27) = 4.54, *p* < .01. However, we did not obtain this pattern of differences in the PCs. The significant differences were associated only with the left-side presentations and the mean PC was greater in trials with low-magnitude digits (92.29%) than with middle-magnitude digits (89.28%), *t*(27) = 2.57, *p* < .05, and high magnitude digits (87.54%), *t*(27) = 3.73, *p* < .01.

### Interaction Between Side of Presentation and Position Factors

The interaction between position and side of stimuli presentation was the result of faster RTs to the digits displayed laterally than to those displayed medially in trials with digits presented on both the right and left side (848 vs. 885 ms, *t*[27] = 2.13, *p* < .05, and 898 vs. 974 ms, *t*[27] = 6.46, *p* < .01, respectively). This pattern of differences seems to be consistent with the main effect of the position factor and the faster retrieval of digits that are presented laterally.

### Interaction Between Side of Presentation, Magnitude, and Position Factors

**Figure 4.**
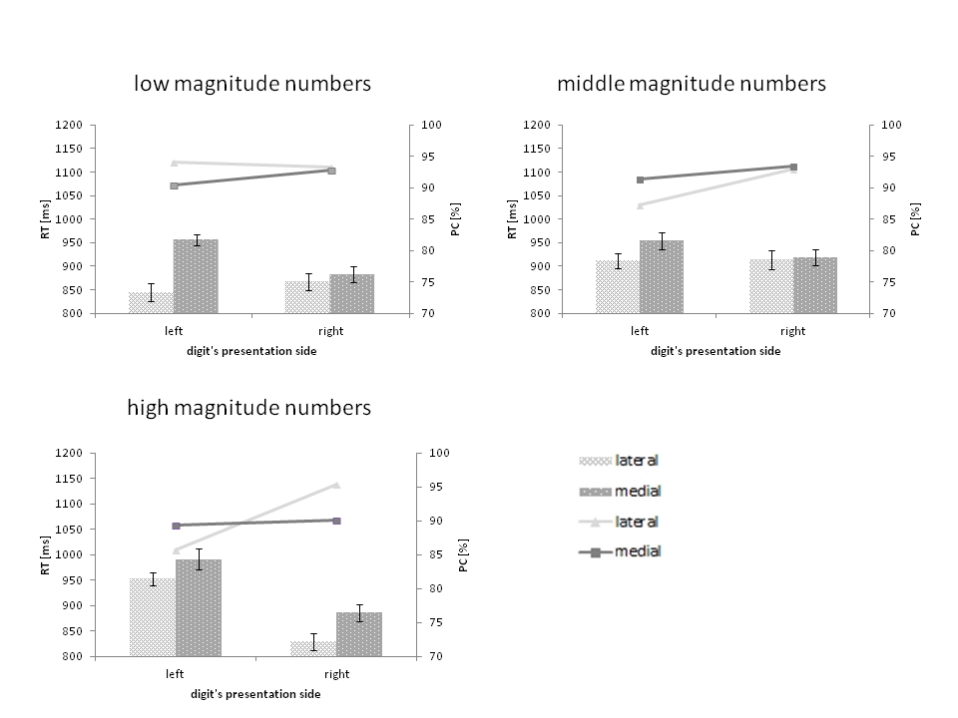
The interaction between the magnitude, position, and side of the digit’s presentation in the mean reaction time (RT; represented by bars) and percentage of correct responses (PC; represented by lines). The error bars represent the CI from normalized data. The values of descriptive statistics illustrated in the figure are presented in Table 1.

In Figure 4, the three-factor interaction between magnitude, position, and side of stimuli presentation for RTs and PCs as dependent variables is illustrated. We found that, when low-magnitude numbers were presented on the left side (congruent trials), the mean RT was significantly shorter for the digits that were displayed more laterally than for those that were displayed more medially, *t*(27) = 6.65, *p* < .01. There was no significant difference in the mean RT for laterally versus medially-presented low numbers when displayed on the right (incongruent trials). However, we obtained a mirrored pattern of results in the trials with high magnitude targets. There was no difference between the RTs for laterally- and medially-presented digits displayed on the left, but for high magnitude numbers that were displayed on the right, the mean RT was shorter for those presented more laterally than for those presented more medially, *t*(27) = 2.54, *p* < .05. The pattern of differences for the numbers 4, 5, and 6 resembled the pattern obtained for low-magnitude numbers-that is, there was no difference between the RTs for laterally versus medially presented numbers displayed on the right. However, there were shorter RTs for laterally presented numbers than for medially presented ones in the case of left side presentation, *t*(27) = 2.56, p < .05.

**Table 1.** Mean and SEM Values Calculated for Reaction Times (RTs) and Percentages Correct responses (PCs), and CIs for RTs in All Experimental Conditions (for Each Side of Presentation, Number Magnitude, and Exact Position of Target)

Number magnitude	Side of presentation	Number position in prime stimuli									
		Lateral					Medial				
		RT			PC		RT			PC	
		*M*	*SEM*	CI	*M*	*SEM*	*M*	*SEM*	CI	*M*	*SEM*
Low	Left	845	33	18.27	94.08	1.5	956	33	12.63	90.5	1.4
	Right	867	29	18.05	93.37	1.53	883	33	17.04	92.86	1.4
Middle	Left	912	30	15.63	87.21	2.68	954	28	18.48	91.35	1.58
	Right	913	32	19.97	92.94	1.42	919	36	16.1	93.52	1.3
High	Left	952	30	12.6	85.66	2.42	991	32	20.71	89.42	1.87
	Right	829	26	16.85	95.47	0.56	886	34	17.18	90.11	1.76

*Note*. Reaction time (RT), percentage of correct responses (PC)

In the trials with neutral numbers, however, there was no significant difference between the mean PCs for both the digits presented on the left and those presented on the right (see Figure 4). The differences between the mean PC in trials with low versus high numbers were consistent with those observed for the RTs. The PCs for low numbers were greater for targets that were displayed laterally on the left than for those that were displayed medially on the left, *t*(27) = 2.44, *p* < .05, whereas the difference between the PCs in the trials with targets presented on the right was not significant. In the case of high magnitude numbers displayed on the right side, there was a greater mean PC for laterally presented targets than for targets presented medially, *t*(27) = 3.71, *p* < .01.

## Discussion

 In this study, we investigated the SNARC effect by asking participants to remember and retrieve stimuli consisting of low and high magnitude numbers as well as middle numbers (mentally positioned in the centre between low and high) that were presented laterally to a central fixation point in four positions, each differing in terms of their side and distance from the central point. This experiment enabled us to answer the question of whether the memorization and retrieval of numerical material from STM is more effective when numbers are presented in a spatial location that is congruent with their positions on the MNL. We observed an influence of STM load (stored with numerical material) on the spatial-numerical association of retrieved material (which confirmed findings from previous studies) and that it was feasible to examine the SNARC effect without the use of an additional task (overloading the memory resources in dual-task procedure) influencing the typical (e.g., parity or magnitude judgment) task performance as it was being developed, for example, in the experiments by Herrera et al. (2008) or Ginsburg and Gevers (2015). Instead, it was investigated directly by a recalling index, and the numbers were not presented in a temporal sequence; all four numbers were presented at the same time in a spatial order from left to right. The obtained results seem to confirm that the SNARC effect in memory task depends, in fact, on the interaction of STM and LTM representations of numbers. The mechanisms of this are likely twofold. First, the MNL representation in LTM influences the time and accuracy of retrieval of numbers with their positions from STM. In other words, congruency between the position of a number in the prime stimulus and its position on the MNL affects the retrieval efficiency. Second, the exact position in the prime stimulus stored in STM is also crucial for the retrieval process (the SNARC effect may be modulated by this). These results are not new and have been revealed in previous studies on this topic. However, the modulation of the SNARC effect by the sequence position of the displayed digits has only been previously investigated by manipulating the temporal order position (as in the study by Ginsburg & Gevers, 2015, or Huber et al., 2016). Moreover, the effect of congruency seems to be different for low and high numbers. Additionally, despite the interaction between magnitude and the presentation side of the digit, we did not reveal a clear SNARC effect because there were no significant differences between congruent and incongruent targets in both low and high numbers. The SNARC effect was only found for the trials with left-sided targets, probably because of the main effect of the magnitude and the exact position factors. The obtained results confirmed several findings indicating the general significance of the side of presentation or attentional processing depending on the numerical magnitude. 

### The Effect of Number Magnitude

One of these findings concerns the effect of number magnitude, as the fastest responses were to low numbers. Thus, 1 and 2 were elicited easier, independent of their position in the stimuli (i.e., also independent of their congruency). However, at the same time, the results obtained for the RTs were not consistent with the greater PCs for low-magnitude numbers. Specifically, there was an effect of magnitude on PC, but the difference in the percentage of correct responses was only significant between middle and high magnitude numbers. This may suggest the involvement of small numbers rather than memory capabilities in driving attention. In fact, this importance of low-magnitude numbers is not surprising when we consider, for one, data from some studies on shifting attention by small numbers, and two, their prevalence in everyday experience.

 Low-magnitude numbers guide our attention, which has been proven by demonstrating an attentional bias slightly towards the left side of the MNL in the numerical intervals bisection task (Göbel, Calabria, Farne, & Rossetti, 2006; Longo & Lourenco, 2007; Longo, Lourenco, & Francisco, 2012). This effect, called *pseudoneglect*, has been established not only for numbers (for a review, see Umiltà, Priftis, & Zorzi, 2009). Schwarz and Eiselt (2009) demonstrated an advantage of low-magnitude numbers in a study on temporal perception of digits. Small numbers were perceived as presented earlier than larger numbers, which was interpreted as the effect of faster processing of smaller numbers. Cai and Li (2015) demonstrated this link between numerical magnitude and spatial attention by testing the ability of small and large numbers to direct attentional focus using a target detection task with the cues composed of pairs of digits. They revealed that targets preceded by low-magnitude numbers had shorter RTs. Our results suggest that this allocation of attention to small numbers may also determine memory processing of numerical material. An effect of magnitude on RT similar to the one obtained here was also shown in Gut et al. ( 2012) , in which the authors used a parity judgment task with low (1 and 2) and high (8 and 9) numbers. On the contrary, Krause, Bekkering, Pratt, and Lindemann (2016) obtained the effect of number magnitude as well. However, the participants in their experiments reacted faster to high (vs. low-) magnitude numbers. The authors employed a visual search task with one-digit numbers, which differed in physical size, numerical magnitude, and color (in Experiment 2), were displayed simultaneously (as in our experiment), and arranged in a circle. Participants were asked to detect the target digit, which differed in physical size from distractors or was displayed in the same size but in a different color. The purpose of their study was to examine the effect of interaction between physical and numerical magnitude on reaction time (the congruity effect). They found that responses were faster to physically larger targets when they were numerically larger, too. Interestingly, they did not observe the same congruity effect for physically small targets. Moreover, they did not obtain the SNARC effect in the relationship between side of response and side of target presentation. What is important, the authors proved that the size congruity effects as well as generally faster RTs to numerically larger numbers were not dependent on the absolute physical size or other perceptual features of the stimuli used in the tasks. Instead, they were dependent on the processing of the number magnitudes of targets. 

 The faster RT associated with low-magnitude numbers (which here is a sign of their faster retrieval) could be described as an effect of the greater ease and automatic nature of processing smaller numbers as well as an effect of our familiarity and more frequent everyday use of the numbers 1 or 2. This influence of familiarity is further proof of the effect of LTM representations on responses to numbers. This finding reflects a well-known relationship-that is, that the more familiar and practiced the stimuli, the more efficient the retrieval of them and, subsequently, the faster the response. This relationship is also consistent with the results of studies using the Random Number Generation (RNG) task; these studies have revealed that individuals produce low-magnitude numbers more frequently than high magnitude ones (Boland & Hutchinson, 2000; Loetscher & Brugger, 2007; Rath, 1966). Some authors (e.g., Loetscher & Brugger, 2007) have claimed that more frequent producing of small numbers is related to the spatial representation of numbers, whereas others (e.g., Rath, 1966) have suggested that this is an effect of the fact that low numbers are learned earlier and they are processed more frequently. On the one hand, this interpretation seems to be in line with findings from studies on numerical competencies in infants, which showed that an ability to discriminate small numbers of objects is observed even in very young individuals (for a review see Dehaene, Dehaene-Lambertz, & Cohen, 1998; Dehaene, Molko, Cohen, & Wilson, 2004; Feigenson, Dehaene, & Spelke, 2004). On the other hand, the latter interpretation could be corroborated, for example, by the results of a study by Loetscher, Schwarz, Schubiger, and Brugger (2008) , who demonstrated more frequent low-number production (via an RNG task) in participants when they were rotating their head to the left, and high number production when they rotated to the right. Nevertheless, some of the data from the literature may contradict this interpretation. Some authors have provided examples of more frequent everyday experiences of dealing with multi-digit numbers (for a review see Nuerk et al., 2011). It has been also reported that decade numbers (10, 20, etc.) have a higher frequency of occurrence in everyday life (Dehaene & Mechler, 1992) and play a special role in development of some mathematical abilities (Siegler & Robinson, 1982). 

 Some authors (see, e.g., Gevers, Ratinckx, De Baene, & Fias, 2006) interpret the differences between RTs to small versus large numbers as being related to the size effect (Buckley & Gillman, 1974), which refers to the greater difficulty in comparing high magnitude numbers than low-magnitude ones (see Cohen Kadosh, Henik, & Rubinsten, 2008). Others (e.g., Dehaene et al., 1993) have noted that faster responses to the digit 2 could be an effect of education practices: When we are taught the definition of parity, we begin learning all even numbers with the number two. Thus, it could be concluded that faster reactions to 1 or 2 may be observed not only by assessing their parity, magnitude, or other features (which is clearly documented in the literature referred to above), but what we proved in our experiment also by retrieval of numerical material from STM. The lack of significant differences between PCs for low- versus high magnitude numbers, which was inconsistent with the pattern of RTs, suggests that participants retrieved low numbers more quickly but not necessarily more correctly. This could be explained in part by the position effect (see below), but it is also possible that although participants generally remembered the side of target presentation, they did not remember whether it was displayed far on the right (or left) or rather on the right (left) but closer to the fixation point. 

### The Effect of the Side of Digit Presentation

 A significant effect was also observed for the side of number presentation, both in RTs and PCs. Retrieval was more effective and efficient when the particular digit was located on the right side of the prime stimulus. This right side predominance is likely also the origin of the SNARC effect only occurring for left-sided presentations (see below). This effect, which in fact indicates an advantage of right-hand responses, may be related to the right-handedness of participants. However, it has been shown that the spatial-numerical association is independent of the hand dominance of participants when they react with both of their hands (Dehaene et al., 1993). Therefore, handedness should not influence the SNARC effect. Furthermore, a clear SNARC effect can also be obtained in tasks using one hand (e.g., Fischer et al., 2003; Santens & Gevers, 2008). Thus, handedness does not seem to be a plausible explanation of these differences in RTs and PCs. The effect of side of presentation may also be related to the functional brain lateralization of attention or memory processing of numbers (e.g., Knops, Nuerk, Fimm, Vohn, & Willmes, 2006). The asymmetry obtained in our results was characterized not only by a bias in RTs and PCs to targets presented on the right side but also by the facilitation of responses to low-magnitude numbers (described above). Some data from the literature have provided evidence of brain asymmetry in number cognition processing. However, this lateralization pattern is task-dependent. For instance, right hemisphere domination has been observed for number comparison and estimation, while the left hemisphere dominates in calculation (see Cohen & Dehaene, 1996), and even other reports have suggested a bilateral control of numerosity estimation (see Dormal, Andres, & Pesenti, 2008). A recent meta-analysis regarding the brain localization of regions engaged in different number tasks and their asymmetry index (Arsalidou & Taylor, 2011) indicated that the general pattern of activations is even more complex. The laterality indices calculated by authors demonstrate that left hemisphere dominance is present in addition, but during subtraction, some areas manifest right- and some left-hemisphere preponderance or bilateral activation, and in multiplication, mainly right-sided regions are engaged. In Gut et al. (2012) , which used event-related potentials, the authors obtained results suggesting that in each hemisphere, the brain mechanisms involved in parity judgment are dependent on the magnitude and congruency of the number used as the target. Namely, in the congruent trials, the left hemisphere seemed to control the cognitive processing of low-magnitude numbers, whereas the right hemisphere supported the same processes with high magnitude numbers (see Salillas, El Yagoubi, & Semenza, 2008, for contrary results). The opposite pattern was found for incongruent trials, in which conflict generated by the numbers activated the hemisphere contralateral to the spatial representation of the number. Based on this inconsistency and complexity in the results from studies on the brain basis of numerical cognition, as well as the use of numerical tasks that differ from those used in our experiment, it is rather difficult to interpret our results in the context of manifestation of a particular brain asymmetry pattern. It should still be considered that in the experimental paradigm developed for this study the task was not an estimation, calculation, or magnitude determination (for which the brain biases have been investigated), but rather to memorize the spatial localization of a digit in a stimulus consisting of a set of numbers. This means that it is not reasonable to directly compare the effects obtained in a numerical memory task with the results from experiments using rather different types of tasks. However, hemispheric lateralization related to such processes is worthy further investigation. The performance of such a memory task should involve the employment of measurements obtained by psychophysiological techniques to provide a further explanation of the presentation side effect that we observed here. 

 It is also plausible that the shorter RTs and greater correctness of retrieval of right-sided targets reflected a recency effect, which is related to STM processing (Deese & Kaufman, 1957). Namely, the digits located on the right side of the stimuli are most likely scanned as the last items because of the left-to-right direction of stimulus examination (see Nachshon, 1985; Nachson & Hatta, 2001) and the effect of direction of object counting preferences on the spatial-numerical association (Opfer, Thompson, & Furlong, 2010 ; Shaki, Fischer, & Göbel, 2012). In fact, it makes this sequence of digits similar to temporal sequences, as in the method used by Ginsburg and Gevers (2015) and in the other studies on the ordinal position effect. Therefore, when the target appears, these recently stored digits are still pronounced and accessible in STM, leading to faster responses and greater correctness of retrieval. 

The pattern of the responses also suggests that for the digits presented on the right, retrieval is equally effective (always faster) independent of the congruity of the stimuli. The profile of the results indicates correspondence between the side effect and the differences observed in the interactions between (1) side and number magnitude and between (2) side and position. The former interaction is manifested in the differences associated with the responses to low/high numbers displayed on the left/right side. Participants responded faster and more correctly to low than to high numbers but only in case of numbers presented on the left side; the same differences for digits displayed on the right were not significant. Thus, it can be concluded that we obtained a clear SNARC effect for the left-sided presentations only and that the effect for right-side presentations became indiscernible. Based on these results, one may conclude that the spatial representations of numbers on the MNL are crucial for retrieval of the numbers presented on the left and that responses to the numbers presented on the right are generally faster and more correct (irrespectively of their congruency). This is consistent with the effect of side independent of congruency. In other words, in the trials in which a target number was presented on the left, it seemed that its congruency determined the responses of the participants. In contrast, in the trials with the target displayed on the right, it did not matter if it was congruent or incongruent. This “one-side” SNARC effect was also visible when studying the responses to high magnitude numbers displayed on the left versus the right side (which is in fact the same as numbers displayed on the congruent vs. incongruent side). We can see that participants needed more time to respond, and that they made more errors during retrieval of high magnitude numbers that were presented on the left (i.e., on the incongruent side) as opposed to the right (i.e., the congruent side). Therefore, this difference seems to be a manifestation of the SNARC effect for high magnitude numbers (the “SNARC effect for one side of the MNL”). In the trials with low-magnitude numbers, the difference between the RTs for left- versus right-presented digits was small, and in fact, an additional analysis revealed that it was not significant, which is consistent with the main effect of magnitude. The specific role of small numbers and their faster processing (described above) may be one of the possible causes explaining this effect.

 However, another explanation of these differences should be considered as well. For one, the RTs to right-side presented digits were faster. In addition, the size of the SNARC effect has been shown to depend on the response latencies. Specifically, the SNARC effect is more pronounced in longer RTs (Gevers, Verguts, Reynvoet, Caessens, & Fias, 2006). Hence, it could be argued that the generally greater latencies of responses to the left-sided targets elicited the SNARC effect, whereas in the trials with right-sided targets this effect was weakened. 

### The Effect of Exact Position in the Stimulus

 Another observed result was the significant effect of the exact position of the target in the prime stimulus and the interactions of this factor with the others. We hypothesized that the spatial-numerical association would be more noticeable in conditions of more lateral presentation, what was based on the relationship between the strength of the SNARC effect and the distance of the processed number from the middle point of the numerical interval (Wood et al., 2008). The results suggest that this may have been true in our study. First, we can see that there were faster responses to numbers displayed on the congruent side, especially in the trials with targets placed in more lateral positions in the prime stimulus, whereas the numbers displayed closer to the fixation point (representing the centre of the one to nine interval) did not evoke a visibly pronounced spatial-numerical relationship. This finding could be described as another “partial” SNARC effect—in this case, the SNARC effect for lateral positions. Second, we also observed that the participants responded faster in general when the targets were displayed more laterally. This finding suggests that when a number is displayed too close to the centre of the numerical interval, the recall and response can be inhibited or that the spatial-numerical association is not sufficiently pronounced. This seems to be related to the distance effect (Moyer & Landauer, 1967; see also Zhang et al., 2016, who obtained the same pattern for names of colors) or maybe (more possibly) to the relationship between the size of the SNARC effect and the number magnitude (Fias et al., 1996). Although the distance effect concerns the magnitude comparison condition, one may consider that in a similar manner it is easier to recall the exact spatial position of the numbers that are located far away from each other, both on the MNL and in the stored stimuli (which applies to the numbers 1, 2, 8, and 9). 

 Thus, our results provide confirmation that the SNARC effect is not just the consequence of the relationship between side of number presentation, motor response and its number magnitude (position of its representation on the MNL), and they support the significance of the distance between processed numbers, as has been shown in many other studies, corroborating the left-right horizontal orientation of the MNL. However, this study additionally found that these effects also affect the digit recall from STM. Other signs of the influence of exact number location were visible in the other interactions. Regarding the medially displayed numbers, it did not matter whether the target had a low-, high, or middle magnitude, which again may suggest that there is no effect of magnitude in trials with targets presented closer to the fixation point. Contrary to this finding, there were differences in the RTs and PCs for laterally presented targets because we obtained clearly faster (and more correct) responses to low numbers in comparison to high and middle ones. It is worth emphasizing that these interactions are in contradiction to the effect observed by Santens and Gevers (2008) , who used one-handed responses with keys located more laterally (labelled *far*) and more medially (defined as *close*) to the central key. They showed that all low-magnitude numbers were associated with close responses and high magnitude ones with far responses. Moreover, it should be noted that this low-close and high-far association was independent of the side (left/right) of the motor reactions, which means, for example, that high magnitude numbers were linked with the far response key even when that key was located on the left side. Our results demonstrated that in the trials with more lateral presentation of numbers (which required far responses) the reactions of the participants were generally faster to congruent than to incongruent targets (but they were not faster for high than for low numbers as these authors reported). This is again confirmation of the importance of the task demands and how expected responses to stimuli were defined. 

When we examine the interaction between the position, side, and magnitude, we can see that the exact position of the number in the stimulus determines the RT and PC only when it is presented on the congruent side. In contrast, for incongruent localization, the differences between the RTs (and PCs) for medially versus laterally presented numbers were not significant. How could these results be considered as a manifestation of an interaction between LTM and STM resources in this type of task performance? For numbers that are stored in STM in positions that are congruent with their mental representations on the MNL (stored in LTM), the exact position matters and modulates the responses of the participants, whereas in the case of incongruent trials, the exact localization of the target number has no effect on RTs and PCs. Additionally, as a consequence, in the trials with low numbers, the differences between the reactions to laterally versus medially displayed targets mirrored the pattern obtained for high numbers. This is another example that the SNARC effect is modulated by the instructions for the current task.

 However, it is interesting that the same factor (position) did not influence the correctness of the response. This observation suggests that it is easier to evoke reactions to laterally presented numbers; however, as a consequence, some of the fast responses may be incorrect. On the one hand, this is a typical consequence of using tasks with RT measurements. However, it should be noted that the effect of competition between RT and correctness has not been reported in studies on the SNARC effect, as RTs are often positively correlated with PCs (Dehaene et al., 1993; Fias et al., 1996). On the other hand, this could be interpreted as indicating that there is a faster preparation of responses to a number when the participant is sure that it was located, for example, on the left side of the prime stimulus but is unsure where exactly—laterally or medially on the left. This means that in the case of numbers displayed on the left responding with the left hand is facilitated, but the reaction may not be compatible with the precise position in the stimulus. Another reason for the lack of an effect in PCs with a coexisting effect on RTs could be, unfortunately, the difference in physical size between the response keys, as the Shift keys are larger than the keys used for responses to medially presented targets. Therefore, the shorter RTs for numbers in a lateral position may simply be a consequence of the shifting of attentional focus towards these keys. Furthermore, the significance of congruency in targets presented laterally may be a manifestation of compatibility between physical size and the number magnitude because of the relationship between the size effect and the distance effect (see Cohen Kadosh et al., 2008) as well as an interference between physical size and numerical magnitude in the magnitude comparison task (Henik & Tzelgov, 1982 ). The potential effect of size of the response keys is worth taking into account in future studies. 

There was one additional finding from the three-factor interaction between magnitude, position, and side of presentation. We can see that in the case of low-magnitude numbers, participants reacted faster and more accurately when the numbers were presented more peripherally on the left than when they were displayed more medially on the left. Generally, presentation of a low number on the left side is a clear example of a congruent condition. However, for each pair of one-digit numbers presented on the same side of the prime stimulus, only one could be low and the other was middle or high (see Method section). Therefore, this would indicate that a low number presented laterally on the left is congruent, and the other number of the pair consequently had to be incongruent. The difference between the mean RTs and PCs for these two numbers in the pair was significant, whereas there were no such differences in trials with small numbers presented on the right (medially or laterally), despite the reverse stimulus pattern. In those situations, the low number presented laterally on the right was incongruent and the other number in the pair had to be congruent. Furthermore, we obtained an exactly mirrored pattern for high magnitude numbers. Therefore, it seems that we have shown two additional manifestations of the SNARC effect revealed within pairs of numbers. We cannot exclude the possibility that this is an effect of a strategy for memorizing the presented stimuli. In other words, it is possible that participants divided the displayed row of numbers into two pairs of one-digit numbers in STM. Thus, within each pair, one of the numbers was on the left and the other was on the right. Consequently, the presentation of a number on its congruent side shortened the RT in the retrieval process particularly when it was displayed laterally on that side. Moreover, this number together with the second one of the pair generated a SNARC effect for half of the memorized prime stimuli. If this is a reflection of a memory strategy, this additional SNARC effect is undoubtedly further proof of the influence of MNL on task performance. However, in addition to the strategy used for STM processing, this issue is worth further examination with control over the use of particular pairs of digits as the stimuli in the same type of task.

 We should consider one more possible strategy of memorizing that could have led to the obtained effects. It is likely that participants facilitated memory processing by grouping the stored material into 2 two-digit numbers. If so, we have to account for the influence of several effects that are attributed to multi-digit numbers that may have affected the task performance as well as some specifics of multi-numbers processing (for a review see Nuerk et al., 2011). On the one hand, the SNARC effect has been reported in two-digit numbers as well (Tlauka, 2002). On the other hand, for example, Zhou, Chen, Chen, and Dong (2008) observed the SNARC effect only for decade digits. In fact, the pattern of distance effect, SNARC effect, and magnitude (size) effect obtained for two-digit numbers is dependent not only on the magnitude of an entire number but also on the magnitude of decade and unit numbers as well as their relationship (Nuerk et al., 2011). It should be stressed that the specificity of multi-digit number processing has been extensively examined for different tasks such as parity judgment, magnitude comparison, number matching, or naming and calculation (for a review see Nuerk et al., 2011), but the role of the mentioned effects in memory processing remains rather unclear. This does not mean, however, that we should rule out the possibility that the way two-digit numbers are processed can affect the memory processing of single one-digit numbers such as those used in our paradigm. The storage and retrieval of digits could be affected by, for example, compatibility between the decade digit (the left of the pair) and the unit digit (the right one, see Nuerk, Weger, & Willmes, 2001, 2002, 2004). In addition, parity effects specific to multi-digit numbers might influence task performance: first, the more effective processing of even numbers (Hines, 1990), and second, the fact that in two-digit numbers the parity of the unit digit defines the parity of the whole number (Reynvoet, Notebaert, & Van den Bussche, 2011). The question of strategies in memorizing and retrieving of numerical information becomes important when we consider that in the task used in our paradigm the participants were asked to remember the spatial position of not one but four digits. Consequently, we are not sure whether there was an effect of the magnitude and congruency of the other 3 one-digit numbers in the prime stimulus (or, at least, the two that are in the pair displayed on the opposite side of the prime stimulus). We can imagine that despite the requirements of retrieving only the target digit, the other numbers are also still processed and, thus, their magnitudes may have interfered with target retrieval. This effect has been confirmed, for example, in a study that used distractor digits that elicited congruent/incongruent response decisions (Nuerk, Bauer, et al., 2005). All of the above-mentioned factors which may be related to possible strategies applicable in the process of storage and retrieval of numbers are worth considering in future studies. Another factor which was not controlled in our procedure but could modify the results is the type of sequence in prime stimuli used in the task (as it was manipulated in the study by Lindemann et al., 2008, who revealed its effect; but see discussion by Huber et al., 2016). It seems relevant to include this variable in future studies on the topic. 

### Shortcomings of the Methodology of the Present Study

 Besides all of the discussed variables potentially influencing the performance in such types of tasks, which should be taken into account during continuation of investigation, there are some limitations to the methodology of our study that need to be emphasized when interpreting the obtained results. One of them could be a small number of trials, which could have influenced the SNARC effect’s strength (e.g., this may be the reason of the lack of main effect of congruency, resulting in partial SNARC effects). The importance of the number of stimuli repetitions has been discussed, for example, by Huber et al. (2016) as well as by Cipora and Nuerk (2013). Another serious limitation, which could affect the profile of the results and which may lead to difficulty in the interpretation of the data, is the lack of mask stimulus or blank screen in the time interval between prime stimulus and target stimulus. As the time of target presentation was relatively long (1 s, see Method section), it cannot be excluded that the image of the prime stimulus was present in sensory STM after its presentation. As a result, it is possible that we measured the effect of STM and visual memory load on the retrieval of digits. However, if this is the case, the question of more effective and efficient retrieval of the digits presented on the right side of prime stimuli becomes more problematic for discussion. 

To sum up, the results obtained in this experiment led to the general conclusion that the retrieval of digits displayed in a spatial sequence from STM can be modulated by LTM representations of numbers organized on the MNL. This impact, however, did not manifest as the SNARC effect, but rather as a demonstration that the SNARC effect can be modulated by factors such as the particular position of a digit in the stimulus or the probable short-term memory strategies used by individuals (e.g., grouping the four digits presented into two-digit numbers or dividing the material into two pairs of digits). Moreover, the process of recalling numbers from STM seemed not to be affected by the increased STM load elicited by an additional task but simply by, for example, attentional shifting induced by number magnitudes, task requirements, or the method of parsing stored numerical material.

## Author Note

This study was supported by Marie Curie ITN-LAN SP3-People (7th Framework Programme, Grant Agreement # 214570). The authors thank M. Ciesielski for his help in programming the experimental paradigm as well as A. Wójcik and M. Wrońska for few but valuable suggestions on the final version of manuscript. The authors also thank the three anonymous reviewers for their helpful comments and suggestions on the manuscript. The experimental paradigm was developed and discussed about six years ago with P. Jaśkowski, who was head of the Psychophysiology lab at that time. This study was one of the final projects that he worked on before he passed away in 2011.

## References

[R1] Arsalidou M., Taylor M. J. (2011). Is 2 + 2 = 4? Meta-analyses of brain areas needed for numbers and
calculations.. Neuroimage.

[R2] Bächtold D., Baumüller M., Brugger P. (1998). Stimulus-response compatibility in representational
space.. Neuropsychologia.

[R3] Ben Nathan M., Shaki S., Salti M., Algom D. (2009). Numbers and space: Associations and
dissociations.. Psychonomic Bulletin & Review.

[R4] Boland P. J., Hutchinson K. (2000). Student selection of random digits.. Journal of the Royal Statistical Society: Series D (The
Statistician).

[R5] Buckley P. B., Gillman C. B. (1974). Comparisons of digits and dot patterns.. Journal of Experimental Psychology.

[R6] Cai Y. C., Li S. X. (2015). Small number preference in guiding attention.. Experimental Brain Research.

[R7] Cipora K., Nuerk H. C. (2013). Is the SNARC effect related to the level of mathematics? No
systematic relationship observed despite more power, more repetitions, and
more direct assessment of arithmetic skill.. Quarterly Journal of Experimental Psychology.

[R8] Cohen L., Dehaene S. (1996). Cerebral networks for number processing: Evidence from a case of
posterior callosal lesion.. Neurocase.

[R9] Cohen Kadosh R., Henik A., Rubinsten O. (2008). Are Arabic and verbal numbers processed in different
ways?. Journal of Experimental Psychology: Learning, Memory and
Cognition.

[R10] Deese J., Kaufman R. A. (1957). Serial effects in recall of unorganized and sequentially
organized verbal material.. Journal of Experimental Psychology.

[R11] Dehaene S. (1992). Varieties of numerical abilities.. Cognition.

[R12] Dehaene S., Bossini S., Giraux P. (1993). The mental representation of parity and number
magnitude.. Journal of Experimental Psychology: General.

[R13] Dehaene S., Dehaene-Lambertz G., Cohen L. (1998). Abstract representations of numbers in the animal and human
brain.. Trends in Neuroscience.

[R14] Dehaene S., Mechler J. (1992). Cross-linguistic regularities in the frequency of number
words.. Cognition.

[R15] Dehaene S., Molko N., Cohen L., Wilson A. J. (2004). Arithmetic and the brain.. Current Opinion in Neurobiology.

[R16] Dormal V., Andres M., Pesenti M. (2008). Dissociation of numerosity and duration processing in the left
intraparietal sulcus: A transcranial magnetic stimulation
study.. Cortex.

[R17] Feigenson L., Dehaene S., Spelke E. (2004). Core systems of number.. Trends in Cognitive Sciences.

[R18] Fias W., Brysbaert M., Geypens F., d’Ydewalle G. (1996). The importance of magnitude information in numeric processing:
Evidence from the SNARC effect.. Mathematical Cognition.

[R19] Fias W., Fischer M. H. (2005). Spatial representation of number. In J. I. D. Campbell (Ed), Handbook of
Mathematical Cognition (pp. 43-54)..

[R20] Fias W., Lauwereyns J., Lammertyn J. (2001). Irrelevant digits affect feature-based attention depending on the
overlap of neural circuits.. Cognitive Brain Research.

[R21] Fias W., van Dijck J. P., Gevers W., Dehaene S., Brannon E. M. (2011). How is number associated with space? The role of working
memory.. Space, time and number in the brain. Searching for the foundations of
mathematical thought.

[R22] Fischer M. H. (2006). The future for SNARC could be stark.. Cortex.

[R23] Fischer M. H., Castel A. D., Dodd M. D., Pratt J. (2003). Perceiving numbers causes spatial shifts of
attention.. Nature Neuroscience.

[R24] Fischer M. H., Shaki S. (2014). Spatial associations in numerical cognition—From single
digits to arithmetic.. The Quarterly Journal of Experimental Psychology.

[R25] Fischer M. H., Shaki S., Cruise A. (2009). It takes just one word to quash a SNARC.. Experimental Psychology.

[R26] Ganor-Stern D., Tzelgov J. (2008). Across-notation automatic numerical processing. Journal of
Experimental Psychology.. Learning, Memory and Cognition.

[R27] Gevers W., Ratinckx E., De Baene W., Fias W. (2006). Further evidence that the SNARC effect is processed along a
dual-route architecture: Evidence from the lateralized readiness
potential.. Experimental Psychology.

[R28] Gevers W., Reynvoet B., Fias W. (2004). The mental representation of ordinal sequences is spatially
organized: Evidence from days of the week.. Cortex.

[R29] Gevers W., Verguts T, Reynvoet B., Caessens B., Fias W. (2006). Numbers and space: A computational model of the SNARC
effect.. Journal of Experimental Psychology: Human Perception and
Performance.

[R30] Ginsburg V., Gevers W. (2015). Spatial coding of ordinal information in short- and long-term memory.
Frontiers in Human Neuroscience, 9..

[R31] Göbel S. M., Calabria M., Farne A., Rossetti Y. (2006). Parietal rTMS distorts the mental number line: Simulating
‘spatial’ neglect in healthy subjects.. Neuropsychologia.

[R32] Gut M., Szumska I., Wasilewska M., Jaśkowski P. (2012). Are low and high number magnitudes processed differently while
resolving the conflict evoked by the SNARC effect?. International Journal of Psychophysiology.

[R33] Henik A., Tzelgov J. (1982). Is three greater than five: The relation between physical and
semantic size in comparison tasks.. Memory and Cognition.

[R34] Herrera A., Macizo P., Semenza C. (2008). The role of working memory in the association between number
magnitude and space.. Acta Psychologica.

[R35] Hines T. M. (1990). An odd effect: Lengthened reaction times for judgments about odd
digits.. Memory and Cognition.

[R36] Hubbard E. M., Piazza M., Pinel P., Dehaene S. (2005). Interactions between number and space in parietal
cortex.. Nature Reviews Neuroscience.

[R37] Huber S., Klein E., Moeller K., Willmes K. (2016). Spatial-numerical and ordinal positional associations coexist in
parallel. Frontiers in Psychology, 7..

[R38] Knops A., Nuerk H. C., Fimm B., Vohn R., Willmes K. (2006). A special role for numbers in working memory? An fMRI
study.. Neuroimage.

[R39] Krause F., Bekkering H., Pratt J., Lindemann O. (2016). Interaction between numbers and size during visual search. Psychological
Research. Advance online publication..

[R40] Lammertyn J., Fias W., Lauwereyns J. (2002). Semantic influences on feature-based attention due to overlap of
neural circuits.. Cortex.

[R41] Lindemann O., Abolafia J. M., Pratt J., Bekkering H. (2008). Coding strategies in number space: Memory requirements influence
spatial-numerical associations.. The Quarterly Journal of Experimental Psychology.

[R42] Loetscher T., Brugger P. (2007). Exploring number space by random digit
generation.. Experimental Brain Research.

[R43] Loetscher T., Schwarz U., Schubiger M., Brugger P. (2008). Head turns bias the brain’s internal random generator. Current
Biology, 18, R60-R62..

[R44] Longo M. R., Lourenco S. F. (2007). Spatial attention and the mental number line: Evidence for
characteristic biases and compression.. Neuropsychologia.

[R45] Longo M. R., Lourenco S. F., Francisco A. (2012). Approaching stimuli bias attention in numerical
space.. Acta Psychologica.

[R46] Morey R. D. (2008). Confidence intervals from normalized data: A
correction to Cousineau (2005). Tutorial in Quantitative Methods for Psychology, 4, 61-64..

[R47] Moyer R. S., Landauer T. K. (1967). Time required for judgments of numerical
inequality.. Nature.

[R48] Nachshon I. (1985). Directional preferences in perception of visual
stimuli.. International Journal of Neuroscience.

[R49] Nachson I., Hatta T. (2001). Directional tendencies of Hebrew, Japanese, and English
readers.. Perceptual and Motor Skills.

[R50] Nuerk H. C., Bauer F., Krummenacher J., Heller D., Willmes K. (2005). The power of mental number line: How the magnitude of unattended
numbers affects performance in an Eriksen task.. Psychology Science.

[R51] Nuerk H. C., Moeller K., Klein E., Willmes K., Fischer M. (2011). Extending the mental number line. A review of multi-digit number
processing.. Journal of Psychology.

[R52] Nuerk H. C., Weger U., Willmes K. (2001). Decade breaks in the mental number line? Putting the tens and units back
in different bins. Cognition, 82, B25-33..

[R53] Nuerk H. C., Weger U., Willmes K. (2002). A unit-decade compatibility effect in German number
words.. Current Psychology Letters: Behavior, Brain and Cognition.

[R54] Nuerk H. C., Weger U., Willmes K. (2004). On the perceptual generality of the unit-decade-compatibility
effect.. Experimental Psychology.

[R55] Nuerk H. C., Wood G., Willmes K. (2005). The universal SNARC effect: The association between number
magnitude and space is amodal.. Experimental Psychology.

[R56] Opfer J. E., Thompson C. A., Furlong E. E. (2010). Early development of spatial numeric associations: Evidence from
spatial and quantitative performance of preschoolers.. Developmental Science.

[R57] Rath G. J. (1966). Randomization by humans.. American Journal of Psychology.

[R58] Reynvoet B., Brysbaert M. (2004). Cross-notation number priming investigated at different stimulus
onset asynchronies in parity and naming tasks.. Experimental Psychology.

[R59] Reynvoet B., Notebaert K., Van den Bussche E. (2011). The processing of two-digit numbers depends on task
instructions.. Journal of Psychology.

[R60] Salillas E., El Yagoubi R., Semenza C. (2008). Sensory and cognitive processes of shifts of spatial attention
induced by numbers: An ERP study.. Cortex.

[R61] Santens S., Gevers W. (2008). The SNARC effect does not imply a mental number
line.. Cognition.

[R62] Schwarz W., Eiselt A. K. (2009). The perception of temporal order along the mental number
line.. Journal of Experimental Psychology: Human Perception and
Performance.

[R63] Shaki S., Fischer M. H. (2008). Reading space into numbers - a cross-linguistic comparison of the
SNARC effect.. Cognition.

[R64] Shaki S., Fischer M. H., Göbel S. (2012). Direction counts: A comparative study of spatially directional
counting biases in cultures with different reading
directions.. Journal of Experimental Child Psychology.

[R65] Siegler R. S., Robinson M. (1982). The development of numerical understandings. In H. W. Reese & L. P.
Lipsett (Eds.), Advances in child development and behavior (Vol. 16, pp.
242-312)..

[R66] Tlauka M. (2002). The processing of numbers in choice reaction
tasks.. Australian Journal of Psychology.

[R67] Umiltà C., Priftis K., Zorzi M. (2009). The spatial representation of numbers: Evidence from neglect and
pseudoneglect.. Experimental Brain Research.

[R68] van Dijck J. P., Fias W. (2011). A working memory account for spatial-numerical
associations.. Cognition.

[R69] van Dijck J. P., Gevers W., Fias W. (2009). Numbers are associated with different types of spatial
information depending on the task.. Cognition.

[R70] Wood G., Nuerk H. C., Willmes K., Fischer M. H. (2008). On the cognitive link between space and number: A meta-analysis
of the SNARC effect.. Psychology Science Quarterly.

[R71] Zhang M., Gao X., Baichen L., Shuyuan Y., Gong T., Jiang T., . . . Chen Y. (2016). Spatial representation of ordinal information. Frontiers in Psychology,
7..

[R72] Zhou X., Chen C., Chen L., Dong Q. (2008). Holistic or compositional representation of two-digit numbers?
Evidence from the distance, magnitude and SNARC effects in a number-matching
task.. Cognition.

